# Flavonoids Identified from Korean *Scutellaria baicalensis* Georgi Inhibit Inflammatory Signaling by Suppressing Activation of NF-**κ**B and MAPK in RAW 264.7 Cells

**DOI:** 10.1155/2013/912031

**Published:** 2013-11-21

**Authors:** Gyeong-Eun Hong, Jin-A. Kim, Arulkumar Nagappan, Silvia Yumnam, Ho-Jeong Lee, Eun-Hee Kim, Won-Sup Lee, Sung-Chul Shin, Hyeon-Soo Park, Gon-Sup Kim

**Affiliations:** ^1^Research Institute of Life Science, BK21 Plus Project, College of Veterinary Medicine, Gyeongsang National University, Gazwa, Jinju 660-701, Republic of Korea; ^2^Department of Physical Therapy, International University of Korea, Jinju 660-759, Republic of Korea; ^3^Korea National Animal Bioresources Bank Laboratory of Biochemistry, College of Veterinary Medicine, Gyeongsang National University, Gazwa, Jinju 660-701, Republic of Korea; ^4^Department of Nursing Science, International University of Korea, Jinju 660-759, Republic of Korea; ^5^Department of Internal Medicine, Institute of Health Sciences, Gyeongsang National University, School of Medicine, Gyeongnam Regional Cancer Center, Gyeongsang National University Hospital, Jinju 660-702, Republic of Korea; ^6^Department of Chemistry, Research Institute of Life Science, Gyeongsang National University, Jinju 660-701, Republic of Korea

## Abstract

*Scutellaria baicalensis* Georgi has been used as traditional medicine for treating inflammatory diseases, hepatitis, tumors, and diarrhea in Asia. Hence, we investigated the anti-inflammatory effect and determined the molecular mechanism of action of flavonoids isolated from Korean *S. baicalensis* G. in lipopolysaccharide- (LPS-) stimulated RAW 264.7 macrophages. A 3-(4,5-dimethylthiazol-2-yl)-2,5-diphenyltetrazolium bromide assay was performed to examine cytotoxicity of the flavonoids at various concentrations of 10, 40, 70, and 100 *µ*g/mL. No cytotoxicity was observed in RAW 264.7 cells at these concentrations. Furthermore, the flavonoids decreased production of inflammatory mediators such as inducible nitric oxide synthase, cyclooxygenase-2, interleukin-6, and tumor necrosis factor-alpha and inhibited phosphorylation of nuclear factor-kappa B (NF-**κ**B) and mitogen-activated protein kinases (MAPKs) in LPS-induced RAW 264.7 cells. Moreover, to identify the differentially expressed proteins in RAW 264.7 cells of the control, LPS-treated, and flavonoid-treated groups, two-dimensional gel electrophoresis and mass spectrometry were conducted. The identified proteins were involved in the inflammatory response and included PRKA anchor protein and heat shock protein 70 kD. These findings suggest that the flavonoids isolated from *S. baicalensis* G. might have anti-inflammatory effects that regulate the expression of inflammatory mediators by inhibiting the NF-**κ**B signaling pathway via the MAPK signaling pathway in RAW 264.7 cells.

## 1. Introduction

*Scutellaria baicalensis *Georgi has been widely used in traditional Chinese herbal medicine to treat various diseases including inflammation, hypertension, cardiovascular disease, and bacterial and viral infections and has officially been listed in the Chinese Pharmacopoeia as a medicinal plant [1]. *S. baicalensis *G. includes four major flavonoids such as baicalin, baicalensis, wogonin, and wogonoside [2], and some studies have reported that baicalin and baicalein have antioxidative and anti-inflammatory effects [3]. 

Inflammation is an important part of immune pathogenesis and is a response to injury, infection, and stress. The acute inflammation reaction is a rapid and self-terminating process; however, if subclinical inflammation continues, it can be harmful to the host as it can be followed by chronic inflammation [4]. During the inflammatory response, diverse mediators such as nitric oxide (NO), prostaglandins (PGs), and proinflammatory cytokines are excessively produced by macrophages [5]. The inflammatory response, including septic shock, fever, and microbial invasion can be initiated by lipopolysaccharide (LPS) which is an endotoxin derived from the cell wall of Gram-negative bacteria [6, 7]. LPS is an influential activator of the immune system and modulates macrophage function [8]. 

PGs are synthesized from arachidonic acid by cyclooxygenase (COX) during the inflammatory reaction, and PGE2 induces fever and pain [9]. Two COX isoenzymes are known which are COX-1 and COX-2. COX-1 is involved in homeostasis and is expressed constitutively in most cells, whereas COX-2 is not produced in normal tissues but is enhanced by oncogenes, growth factors, and cytokines [5]. NO, a free radical produced by nitric oxide synthase (NOS) from L-arginine, is an important cellular signaling molecule. Inducible NOS (iNOS) that produces considerable NO is involved in the immune response and plays a key role in the innate immune response to infectious diseases [10]. 

Several etiologic inflammatory mediators including the cytokines TNF-*α*, IL-6, and IL-1*β* are chronically elevated by persistent exposure to certain microbial pathogens, which lead to inflammatory disease [11]. These cytokines play a role in the host immune response against intracellular pathogens in murine and human models [12]. During the inflammatory response, TNF-*α*, IL-6, and IL-1*β* cause fever by initiating metabolic changes in the hypothalamic thermoregulatory center.

NF-*κ*B is an essential transcription factor that regulates pro-inflammatory gene expression such as iNOS, COX-2, tumor necrosis factor (TNF)-*α*, interleukin (IL)-1*β*, and IL-6 [13]. Under normal conditions, NF-*κ*B exists as a hetero or homodimer and is suppressed by the I*κ*B*α* and I*κ*B*β* inhibitory proteins in the cytoplasm of nonstimulated cells [14]. Some stimulus signals activate I*κ*B kinases, and then the NF-*κ*B complex is free to enter the nucleus, bind to target sites, and upregulate transcription of specific genes involved in the inflammatory response [8]. 

MAPKs are part of a signaling cascade that initiates inflammatory cellular responses to a variety of extracellular stimuli [15]. In mammals, three major MAPKs subfamilies have been described such as ERK, JNK, and p38 MAPK [16]. Secretion of several macrophage factors such as TNF-*α*, IL-1, IL-6, and NO requires MAPK activity [8]. 

The objective of this study was to investigate the anti-inflammatory effects of the flavonoids isolated from Korean *S. baicalensis *G. on the expression of iNOS, COX-2, IL-6, and TNF-*α* via blocking of the NF-*κ*B and MAPK signaling pathways in LPS-stimulated RAW 264.7 macrophages. We studied the expression changes of inflammatory mediators (iNOS, COX-2) and proinflammatory cytokines (IL-6, TNF-*α*) at the mRNA and protein levels as well as I-*κ*B, NF-*κ*B, and MAPK activity in LPS-induced RAW 264.7 macrophages in response to flavonoids isolated from Korean *S. baicalensis*. In addition, we analyzed the differentially expressed proteins in response to LPS and the effect of the flavonoids from Korean *S. baicalensis *G. on RAW 264.7 macrophages using two-dimensional electrophoresis (2-DE) and matrix assisted laser desorption/ionization time of flight mass spectrometry (MALDI-TOF/MS). 

## 2. Materials and Methods

### 2.1. Cell Culture and Reagents

Mouse RAW 264.7 macrophages (Korea Cell Line Bank, Seoul, Korea) were grown in an atmosphere of 5% CO_2_ at 37°C. The cells were maintained in Dulbecco's modified Eagle's medium (Hyclone, Logan, UT, USA) containing 10% heat-inactivated fetal bovine serum, 100 U/mL penicillin, and 100 *μ*g/*μ*L streptomycin (Gibco, Grand Island, NY, USA). LPS from *Escherichia coli *O111:B4 and 3-(4,5-dimethylthiazol-2-yl)-2,5-diphenyltetrazolium bromide (MTT) were purchased from Sigma-Aldrich (St. Louis, MO, USA). A protease inhibitor cocktail kit was purchased from Thermo Scientific (Pittsburgh, PA, USA). Antibodies to COX-2, iNOS, and PCNA were obtained from Santa Cruz Biotechnology (Santa Cruz, CA, USA). *β*-actin, p65, and p50 antibodies were purchased from Millipore (Billerica, MA, USA). NF-*κ*B, I*κ*B, p-I*κ*B, total-ERK, total-JNK, total-p38 MAPK, p-ERK, p-JNK, and p-p38 MAPK antibodies were purchased from Cell Signaling Technologies (Beverly, MA, USA). All other materials and chemicals used were purchased from Ameresco Inc. (Solon, OH, USA) and Sigma Chemical Co. and were of the highest quality commercially available.

### 2.2. Isolation of Flavonoids from Korean *S. baicalensis* G. and Identification by High-Performance Liquid Chromatography-Tandem Mass Spectrometry (HPLC-MS/MS)

*S. baicalensis*. G. radix was obtained from Animal Bioresources Bank (Jinju, Korea). A total of 100 g of lyophilized sample was grounded and extracted with 1000 mL of 70% methanol for 24 h at 50°C. After being evaporated with an Eyela NVC-2100 rotary evaporator (Tokyo Rikakikai, Tokyo, Japan), the enriched solution was extracted three times with n-hexane and ethyl acetate. The organic solvent was collected and yielded 0.6 g of flavonoids. The flavonoids were stored at −20°C until use. HPLC-MS/MS was conducted with an Agilent 100 series liquid chromatograph system (Agilent Technologies, Palo Alto, CA, USA). The chromatographic separation was performed on an Analytical SB-C18 column (4.6 × 250 mm, 5 *μ*m, Agilent Technologies). The flavonoids isolated from Korean* S. baicalensis *G. were separated and identified according to their retention times with 0.1% aqueous formic acid—methanol : acetonitrile (1 : 1, v/v) as the mobile phase in a linear gradient elution. The flow rate was 0.5 mL/min, and column temperature was maintained at 30°C. The HPLC spectra were examined at wavelengths of 240–600 nm and 2 nm resolutions for each test run. Chromatographic results were collected and manipulated with Chemstation, Rev.B.0302. The flavonoids were examined by extracting the chromatograms at 280 nm. MS/MS analyses were performed on a 3200 Q TRAP LC -MS/MS system (Applied Biosystems, Foster City, CA, USA). The mass spectra were recorded over *m*/*z* 100–1500 with a step of 0.1 amu.

### 2.3. Evaluation of Cell Viability

RAW 264.7 cells were cultured in 12-well plates and incubated overnight. The cells were pretreated with 10, 40, 70, and 100 *μ*g/mL flavonoids for 1 h and then treated with LPS (1 *μ*g/mL) for 24 h. The cells were incubated in 100 *μ*L MTT solution (5 mg/mL in phosphate-buffered saline; PBS) at 37°C for 3 h. The violet crystals were dissolved with 500 *μ*L dimethyl sulfoxide. Absorbance of the solution was read at 540 nm using an enzyme-linked immunosorbent assay microplate reader. Cell viability was expressed as a percentage, and the experiment was conducted in triplicate.

### 2.4. Free Radical Activity

DPPH free radical activity of the flavonoids derived from Korean *S. baicalensis*. G. was determined using the stable 1,1-diphenyl- 2-picrylhydrazyl radical (DPPH). Concentrations of 10, 40, 70, and 100 *μ*g/mL flavonoids were mixed with a methanol solution of DPPH (100 *μ*M) in a 96-well microtiter plate. The mixtures were incubated for 30 min in the dark at room temperature, and absorbance was recorded at 517 nm. The percentage of remaining DPPH free radical activity was calculated with the following formula:

(1) $$\begin{array}{c} \text{remaining}\;\text{DPPH}\;\text{activity}\;\;\left(\%\right)=\left(A_{1}/A_{0}\right)\times 100, \end{array}$$

where *A*_0_ is the absorbance of the control and *A*_1_ is the absorbance of the sample and standards.

### 2.5. Enzyme-Linked Immunosorbent Assay (ELISA)

To investigate the effect of Korean *S. baicalensis* G. on cytokine production from LPS-stimulated cells, RAW 264.7 cells were seeded at 2 × 10^6^ cells into 60 mm culture dishes and pretreated with 10, 40, 70, and 100 *μ*g/mL flavonoids for 1 h prior to treatment with 1 *μ*g/mL of LPS for 24 h. TNF-*α* and IL-6 released from treated RAW 264.7 macrophages cells were measured in cell culture supernatants using a commercial TNF-a (mouse) and IL-6 (mouse), EIA kit (Enzo Life Sciences), respectively, according to the manufacturer's protocol.

### 2.6. Western Blot Analysis

Cells were washed twice with ice-cold PBS, and lysates were prepared by suspending the cells in lysis buffer (50 mM Tris-HCl; pH 8.0, 150 mM NaCl, 0.5% sodium deoxycholate, 0.1% sodium dodecyl sulfate (SDS), 1% NP-40, protease inhibitor cocktail, 0.5 M EDTA, and phosphatase inhibitor). Total protein concentrations in each lysate were determined with a Bradford assay kit (Bio-Rad, Hercules, CA, USA). The cell lysates were separated by 10% SDS-polyacrylamide gel electrophoresis (SDS-PAGE) and were transferred to polyvinylidene fluoride membranes (Immobilon-P, 0.45 mm; Millipore, Billerica, MA, USA). The membranes were blocked with 5% fat-free skim milk at room temperature for 30 min and then incubated with iNOS, COX-2, NF-*κ*B, total-ERK, total-JNK, total-p38 MAPK, phospho-I*κ*B, phospho-ERK, phospho-JNK, phospho-p38 MAPK, PCNA, and *β*-actin antibodies overnight at 4°C. The membranes were incubated with secondary antibody at a 1 : 1,000 dilution for 2 h. The immunoblot was visualized by enhanced chemiluminescence (GE Healthcare Life Sciences, Buckinghamshire, UK) and developed on X-ray film (Fuji, Tokyo, Japan).

### 2.7. Reverse Transcription-Polymerase Chain Reaction (RT-PCR)

Total RNA was extracted from RAW 264.7 cells using Trizol reagent (GeneALL Biotechnology, Seoul, Korea). Reverse transcription and cDNA synthesis were performed using a first-strand cDNA synthesis kit (iScript cDNA Synthesis Kit; Bio-Rad) according to the manufacturer's protocol. Total RNA for RT PCR was assessed using specific primers, and PCR cycles were repeated 30–32 times under the following conditions. The RT-PCR conditions were the same as those described previously [17]. COX-2 (sense 5′-CCCAGAGCTCCTTTTCAACC-3′, antisense 5′-ATTTGGCACATTTCTTCCCC-3′), iNOS (sense 5′-CTCCCCTCTCTCCCTTTCCT-3′, antisense 5′-TGGAAATTGGGGTAGGAAGG-3′), TNF-a (Sense 5′-AGCACAGAAAGCATGATCCG-3′, antisense 5′-GTTTGCTACGACGTGGGCTA-3′), IL-6 (sense 5′-CGATGATGCACTTGCAGAAA-3′, antisense 5′-TGGAAATTGGGGTAGGAAGG-3′), and GAPDH (sense 5′-AAGGGTCATCATCTCTGCCC-3′, antisense 5′-GTGATGGCATGGACTGTGGT-3′). The final PCR products were stained with ethidium bromide and electrophoresed on a 1.5% agarose gel. The amount of mRNA was evaluated by Image J software. A quantitative analysis was performed to compare the signal intensity of the specific mRNAs and GAPDH as the internal control.

### 2.8. Protein Extraction for 2-DE

Total protein was extracted from RAW 264.7 cells of the control, LPS-treated, and flavonoid-treated groups. Briefly, 0.15 g of the cell pellet was dissolved in 500 *μ*L of lysis buffer containing 7 M urea, 2 M thiourea, and 4% (w/v) CHAPS. After a 10 min sonication, the samples were centrifuged at 15000 rpm and 4°C for 30 min, and the supernatant was collected. A 10 *μ*L aliquot of the supernatant was treated with 20% TCA (v/v) and incubated at −20°C for 3 h to precipitate the protein. The samples were then centrifuged at 15000 rpm and 4°C for 10 min, and the supernatant was discarded. The protein pellets were dried in a vacuum lyophilizer, dissolved in 500 *μ*L of the lysis buffer, and centrifuged at 15000 rpm and 4°C for 30 min. The supernatant was transferred to new tube and stored at −80°C until needed. Protein concentration was estimated using a noninterfering protein assay kit (Biosciences, St. Louis, MO, USA) according to the manufacturer's instructions.

### 2.9. 2-DE

A total of 200 *μ*g of protein from each sample was applied to an IPG strip in the first dimension (Immobiline DryStrip, pH 4–7NL, 18 cm, GE Healthcare Life Sciences) for the IEF process, followed by 200 V for 1 h, 500 V for 30 min, 4000 V for 30 min, 4000 V for 1 h, 10000 V for 1 h, 10000 V for 12 h, and 50 V for 3 h. The protein samples were focused for a total of 100.2 kVh. The strips were equilibrated the first time with 10 mg/mL DTT in an equilibration buffer for 15 min and the second time with iodoacetamide for 15 min with continuous shaking. In the second dimension, the equilibrated strips were placed onto 12% SDS-PAGE and run at a constant 15 mA until the dye reached the bottom of the gel. The protein spots in the analytical gels were visualized by silver staining. 

### 2.10. Image and Data Analysis

The gel images were acquired using an EPSON V700 photo scanner and imported into Progenesis SameSpots 2D image ver. 4.1 software (Nonlinear Dynamics, Newcastle, UK) for analysis. Protein spots showing more than a 1.5-fold (*P* < 0.05) change in abundance or expression were considered differentially expressed. All spots were confirmed visually and edited manually.

### 2.11. Identification of Differentially Expressed Proteins by MS

The selected protein spots were excised from the 2-DE gel for identification. In-gel digestion of the selected protein spots on the gels was performed as described by [18] with minor modifications. The excised protein spots were proteolyzed in-gel with porcine trypsin. The tryptic fragment masses were detected by MALDI-TOF MS using a Perceptive Biosystems Voyager-DE STR mass spectrometer. The proteins were identified using a Mascot-Peptide Mass Fingerprint (http://www.matrixscience.com/) database search. The following parameters were used for the database searches: taxonomy, mammalians, cleavage specificity, trypsin with one missed cleavage allowed, peptide tolerance of 50 ppm for fragment ions, allowed modifications, Cys carbamidomethyl (fixed), and oxidation of Met (variable). The MOWSE score and species were considered to identify the correct protein from the mascot results list.

### 2.12. Statistical Analysis

Values are expressed as mean ± standard deviation of at least three independent values for each experiment. A Student's *t*-test was performed with SPSS ver. 10.0 for Windows (SPSS Inc., Chicago, IL, USA) that was used to identify differences. A *P* < 0.05 was considered significant.

## 3. Results

### 3.1. LC Chromatogram of Flavonoids Isolated from Korean *S. baicalensis* G.

**Figure 1:**
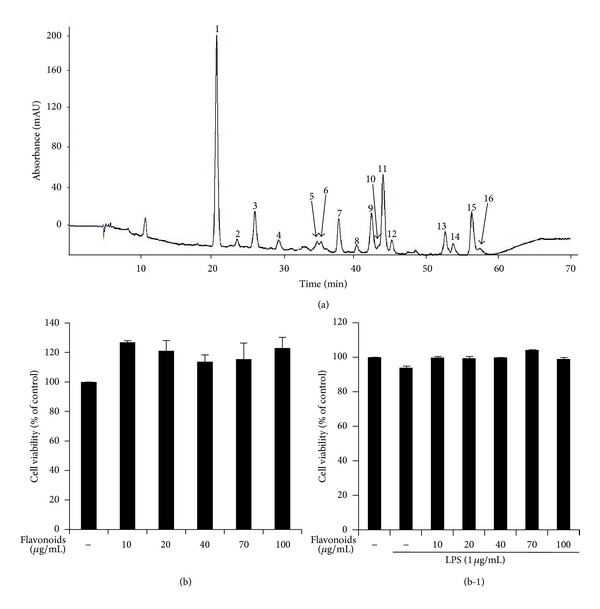
(a) High performance liquid chromatography profiles at 280 nm of the polyphenolic extract from Korean *Scutellaria baicalensis* G.: (1) pentahydroxyflavanone derivative, (2) pentahydroxyflavanone, (3) viscidulin I –O– diglucoside, (4) pentahydroxyflavone, (5) unidentified, (6) viscidulin III–O– glucoside, (7) tetrahydroxyflavone, (8) iridin, (9) eriodictyol (4′-hydroxynaringenin), (10) puerarin, (11) viscidulin III, (12) pentahydroxyflavone, (13) unidentified, (14) baicalin, (15) scutellarein, and (16) isoscutellarein, and (b) the effect of the flavonoids on RAW 264.7 macrophage viability was investigated by MTT assay at various concentrations (10, 40, 70, and 100 *μ*g/mL) in the absence (b) or presence of lipopolysaccharide (1 *μ*g/mL) (b-1).

Flavonoids were isolated from Korean *S. baicalensis* G. using HPLC at the Department of Chemistry, Gyeongsang National University, by Professor Shin, Sung Chul. Sixteen peaks were presented and identified in Korean *S. baicalensis* G. based on the HPLC retention time characteristics and the ultraviolet-visible spectra of standard compounds in a library (Figure 1(a)).

### 3.2. Cytotoxic Effects of Flavonoids on RAW 264.7 Cells

To determine the cytotoxic effects of the flavonoids, cell viability was evaluated at various concentrations of flavonoids (10, 40, 70, and 100 *μ*g/mL) by the MTT assay after incubating the cells for 24 h in the presence or absence of LPS (1 *μ*g/mL). The results showed that LPS (1 *μ*g/mL) and flavonoids of 10–100 *μ*g/mL were not cytotoxic to RAW 264.7 cells. Therefore, flavonoids concentrations up to 100 *μ*g/mL were used for subsequent experiments (Figures 1(b) and 1(b-1)). 

### 3.3. Effects of Flavonoids on RAW 264.7 Cell Morphology

**Figure 2:**
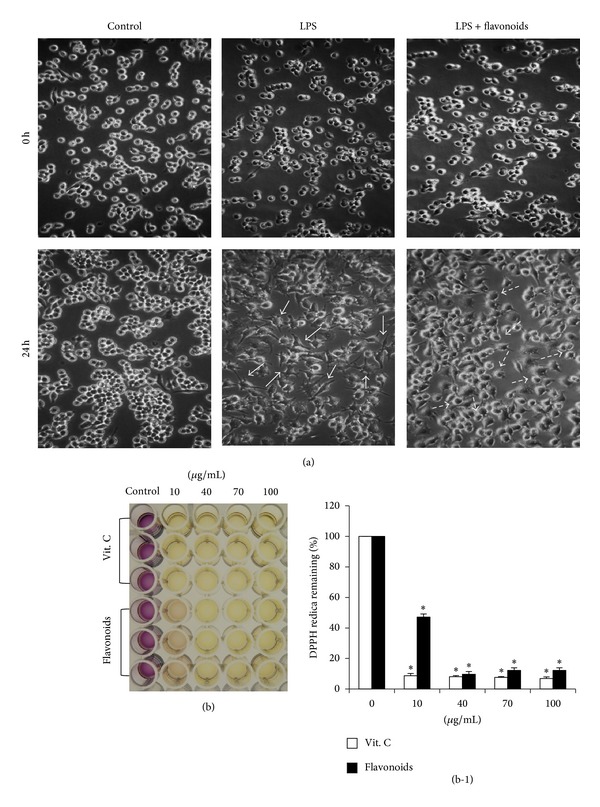
(a) The morphology change of RAW 264.7 cells visualized by optical microscopy (×400). Arrow bars indicate lipopolysaccharide-induced activated RAW 264.7 cells and dotted arrows indicate restored Raw 264.7 cells with the flavonoids. (b) Antioxidative effects of flavonoids: DPPH radical remaining after adding the flavonoids was evaluated by the DPPH assay. The cells were pretreated for 1 h with the indicated concentrations of flavonoids, followed by stimulation with LPS (1 *μ*g/mL) for 24 h. Data shown are the mean ± standard deviation. Three independent experiments were conducted, and differences between mean values were assessed by Student's *t*-test. *P < 0.05.

Figure 2(a) shows the changes in RAW 264.7 cell morphology following flavonoid treatment in the presence or absence of LPS (1 *μ*g/mL). The cells were monitored through an optical microscope with ×400 magnification after a 24 h incubation. Normal cell morphology was generally of round, smooth, and uniform shape, whereas LPS-activated RAW 264.7 cells were irregular and rough with accelerated spreading and formation of pseudopodia. Cotreatment of LPS with the flavonoids reduced the level of cell spreading and pseudopodia formation in a dose-dependent manner.

### 3.4. Remaining Free-Radical Activity

The remaining free-radical activity of the flavonoids was determined using the DPPH method. Vitamin C was used as the positive control and showed 8.4, 7.7, 7.0, and 6.5% of remaining free-radical activity at 10, 40, 70, and 100 *μ*g/mL flavonoids, respectively (Figure 4). The remaining DPPH radical activity values of the flavonoids isolated from Korean *S. baicalensis* G. were 47, 10, 11, and 12% at 10, 40, 70, and 100 *μ*g/mL, respectively (Figures 2(b) and 2(b-1)). 

### 3.5. Effects of the Flavonoids on COX-2 and iNOS Expression in RAW 264.7 Cells

**Figure 3:**
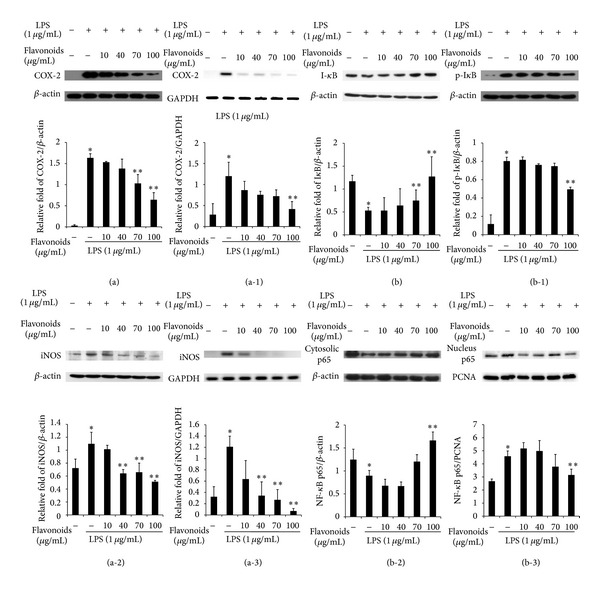
The inhibitory effect of flavonoids on expression of proinflammatory mediators such as cyclooxygenase-2 (COX-2) protein (a), COX-2 mRNA (a-1), inducible nitric oxide synthase (iNOS) protein (a-2), and iNOS mRNA (a-3), and (b) the effect of *Scutellaria baicalensis* flavonoids on NF-*κ*B signal pathway was determined by Western blot assay. The cells were pretreated for 1 h with the indicated concentrations of flavonoids, followed by stimulation with LPS (1 *μ*g/mL) for 24 h and 6 h in protein and mRNA, respectively. In NF- *κ*B signal pathway analysis, RAW 264.7 cells were pretreated for 1 h with the indicated concentrations of flavonoids, followed by stimulation with LPS (1 *μ*g/mL) for 30 min. Data shown are the mean ± standard deviation. Three independent experiments and differences between mean values were assessed by Student's *t*-test. (∗) indicates an increased pattern relative to the control (*P* < 0.05), and (∗∗) indicates a decreased pattern relative to the lipopolysaccharide (LPS) group (*P* < 0.05).

The effects of the flavonoids on COX-2 and iNOS mRNA and protein expression in RAW 264.7 cells were determined by RT-PCR and Western blot, respectively. RAW 264.7 cells activated with LPS had high COX-2 and iNOS mRNA and protein levels when compared with those of the control. COX-2 and iNOS mRNA and protein expression decreased significantly in RAW 264.7 cells after flavonoid treatment (Figures 3(a) to 3(a-3)). These results indicate that LPS-induced COX-2 and iNOS mRNA and protein levels were suppressed in RAW 264.7 cells treated with flavonoids isolated from Korean *S. baicalensis* G.

### 3.6. Phosphorylation and Degradation of I*κ*B and Translocation of NF-*κ*B p65 into the Nucleus in RAW 264.7 Cells Pretreated with Flavonoids

We investigated the effects of the flavonoids on LPS-induced degradation and phosphorylation of I*κ*B by Western blot analysis. I*κ*B level decreased significantly following LPS-stimulation, but flavonoids increased the I*κ*B protein level in a dose-dependent manner (Figure 3(b)). In contrast, LPS-induced phosphorylation of I*κ*B and phospho-I*κ*B was degraded by flavonoids at various concentrations (10, 40, 70, and 100 *μ*g/mL) in RAW 264.7 cells (Figure 3(b-1)). The roles of the transcription factor NF-*κ*B were investigated in the LPS-induced responses. NF-*κ*B was activated when I*κ*B was inhibited in the phosphorylated form, and translocation of p65 to the nucleus increased after LPS induction. However, p65 translocation into the nuclear fraction was suppressed by the flavonoids, whereas p65 increased in the cytoplasm fraction following the flavonoid treatment. These results indicate that the flavonoids isolated from Korean *S. baicalensis* G. increased I*κ*B protein level by dephosphorylating I*κ*B and translocating p65 to the cytoplasm from the nucleus in LPS-induced RAW 264.7 cells (Figures 3(b-2) and 3(b-3)). 

### 3.7. Flavonoids Suppress the LPS-Induced MAPK Pathway

**Figure 4:**
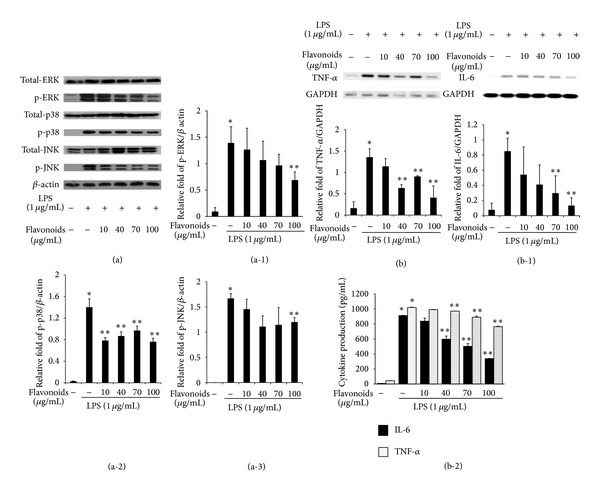
(a) The flavonoids suppressed the activation of MAPK signaling pathway. (b) The mRNA expression of proinflammatory cytokines TNF-*α*, (b-1) IL-6, and (b-2) protein expression of TNF-*α* and IL-6. Raw 264.7 cells were pretreated for 1 h with the indicated concentrations of flavonoids, followed by stimulation with LPS (1 *μ*g/mL) for 15 min, 6 h, and 24 h in MAPK signaling pathway assay, mRNA assay, and ELISA, respectively. Data shown are the mean ± standard deviation. Three independent experiments were conducted, and differences between mean values were assessed by Student's *t*-test. (∗) indicates an increase in expression relative to the control (*P* < 0.05), and (∗∗) indicates a decrease in expression relative to the lipopolysaccharide (LPS) group (*P* < 0.05).

MAPKs are important mediators involved in cellular responses to stressful stimuli. To assess whether inhibition of the inflammatory response by flavonoids is moderated by the MAPK pathway, we examined the effect of the flavonoids on ERK, JNK, and p38 phosphorylation in LPS-induced RAW 264.7 cells. ERK, JNK, and p38 phosphorylation were strongly promoted by LPS (1 *μ*g/*μ*L) and the flavonoids significantly inhibited ERK, JNK, and p38 phosphorylation whereas total forms of ERK, JNK, and p38 were maintained at various concentrations (10, 40, 70, and 100 *μ*g/mL) in RAW 264.7 cells (Figures 4(a) to 4(a-3)). These results show that the flavonoids isolated from Korean *S. baicalensis* G. suppressed the LPS-induced MAPK pathway by inhibiting ERK, JNK, and p38 phosphorylation in LPS-induced RAW 264.7 cells. 

### 3.8. Inhibition of Cytokine Expression by the Flavonoids

The flavonoids inhibited proinflammatory cytokines in LPS-induced RAW 264.7 cells. RT-PCR analyses and ELSIA were conducted to determine whether pretreatment with the flavonoids impacted production of inflammatory cytokines such as IL-6 and TNF-*α*. The levels of the cytokines increased significantly following LPS treatment in comparison with those in the untreated control group. TNF-*α* and IL-6 expression decreased in LPS-induced RAW 264.7 cells treated with the flavonoids in both mRNA and protein level (Figures 4(b) to 4(b-2)). This result shows that flavonoids isolated from Korean *S. baicalensis* G. suppressed cytokines at the mRNA transcription and protein expression in LPS-induced RAW 264.7 cells in a dose-dependent manner.

### 3.9. 2-DE and MALDI-TOF/MS

**Figure 5:**
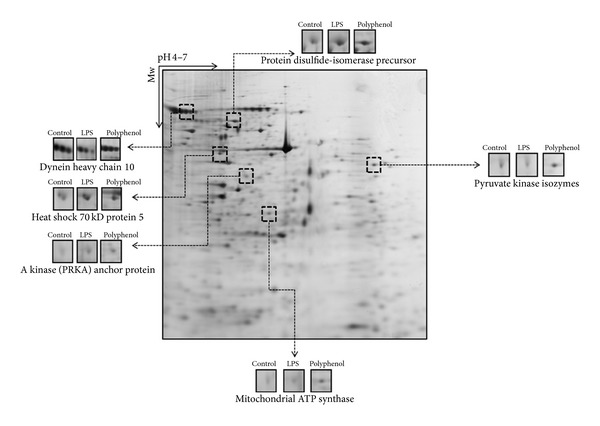
The differentially expressed two-dimensional electrophoresis (2-DE) patterns of RAW 264.7 murine macrophage cells were visualized. The sample was resolved by 2-DE on pH 4–7 IPG strips followed by separation on 12% sodium dodecyl sulfate-polyacrylamide gel electrophoresis in the second dimension. The proteins were visualized by silver nitrate staining.

Proteins from the RAW 264.7 cells in the control, LPS-treated, and flavonoid-treated groups were extracted and resolved by 2-DE using pH 4–7 IPG strips loaded with 200 *μ*g of total protein. The molecular weights of the spots were 17–175 kDa. Differences in spot density were identified as quantitative changes. Ten differentially expressed protein spots were identified (more than twofold was considered significant) (Figure 5), and six proteins were detected by MALDI-TOF/MS. The identified proteins are listed in Table 1.

## 4. Discussion

Different plant extracts have been used as medicine for treating a wide variety of disorders including acute and chronic inflammation. Various plants produce flavonoids in high quantity [19]. Flavonoids are naturally occurring botanical polyphenols whose major role in plants is to act as natural antioxidants [15]. Flavonoids have medicinal and pharmacological activities against inflammation, allergies, viruses, cancer, and other ailments [20]. 

*S. baicalensis *G. has been used as traditional herbal medicine in Asian countries for inflammatory diseases, hyperlipidemia, arteriosclerosis, and some other diseases [21]. The purpose of this study was to evaluate the anti-inflammatory effects and determine the molecular mechanism of a *S. baicalensis* extract in LPS-stimulated RAW 264.7 cells. 

The phenolic contents in the *S. baicalensis* extract were identified by HPLC. Figure 1(a) shows the HPLC profile of the *S. baicalensis *G. extract, which included several flavonoids. The MTT assay results showed that the *S. baicalensis *G. extract was not cytotoxic and had no effect on RAW 264.7 cell viability at concentrations of 10, 40, 70, and 100 *μ*g/mL (Figures 1(b) and 1(b-1)). To examine the effect of the *S. baicalensis* flavonoids on morphological changes of LPS-stimulated RAW 264.7 macrophage cells, microscopic observations were taken at ×400 magnification. Activation of macrophages was expressed as increased cell size and cytoplasmic spreading. Additional changes in activated macrophages affect the immune response [22]. Figure 2(a) shows that normal RAW 264.7 cells were uniform in shape, round, and smooth but became irregular in shape with spreading and pseudopodia after being activated by LPS. Adding the *S. baicalensis *flavonoids reduced the degree of cell spreading and pseudopodia formation. The results of this morphological study demonstrate that *S. baicalensis *flavonoids suppressed cell activation.

The imbalance of excessive reactive oxygen species is called oxidative stress, which can arise from endogenous or exogenous sources and can cause many diseases, including arthritis, sepsis, and inflammatory bowel disease. Antioxidative activity plays an important role as a cytoprotectant by restraining and scavenging radicals or by promoting their decomposition [15]. The antioxidative capacity of *S. baicalensis *G. flavonoids was investigated and verified by the DPPH assay. Phenolic OH is indispensable for antioxidative and free-radical scavenging activity. Figures 2(b) and 2(b-1) show that the *S. baicalensis *extract retained significant activity for scavenging free radicals indicating that the extract of *S. baicalensis *G. consisted of polyphenolic chemicals including hydrogen donors.

COX-2 and iNOS produce large amounts of proinflammatory mediators during the inflammatory process [6]. COX-2 and iNOS expression is associated with the inflammatory response and is induced by inflammatory stimuli, hormones, and growth factors leading to activation of cell signaling [5, 8, 23]. We found that the *S. baicalensis *G. flavonoids exhibited inhibitory effects on iNOS and COX-2 protein expression in a dose-dependent manner in the Western blot analysis. Furthermore, we ascertained that flavonoids also suppressed COX-2 and iNOS mRNA expression by RT-PCR (Figures 3(a)–3(a-3)).

In mammals, the expression of a number of immunity and inflammation related genes such as iNOS, COX-2, TNF-*α*, IL-1*β*, and IL-6 is modulated by activated NF-*κ*B [6]. In normal cells, NF-*κ*B is principally comprised of p50 and p65 subunits bound to inhibitory I*κ*Bs within the cytoplasm [24]. After induction by immunostimulatory ligands, I*κ*Bs are promptly phosphorylated and degraded via the action of I*κ*B kinase (IKK) complex, and the freed NF-*κ*B migrates to the nucleus [1, 6]. Most anti-inflammatory drugs interrupt the expression of proinflammatory genes by inhibiting the NF-*κ*B activation pathway. To investigate the preventive effect of flavonoids on NF-*κ*B activation, we studied p65 nuclear translocation by Western blot analysis. Figures 3(b) to 3(b-3) show that the flavonoids significantly reduced phosphorylation of I*κ*Bs and that p65 translocation decreased subsequently in a dose-dependent manner. These results indicate that the flavonoids derived from Korean *S. baicalensis *G. possess anti-inflammatory activity by suppressing NF-*κ*B activation.

I*κ*Bs are phosphorylated by activating MAPKs and PI3K/Akt [24]. MAPKs are part of a signaling cascade where various extracellular stimuli converge to initiate cellular responses including gene expression, mitosis, differentiation, and cell survival/apoptosis. In mammals, MAPKs include more than a dozen enzymes and ERK1/2, JNK1 to 3, and p38 are very well known. LPS activates all three types of MAPKs in mouse macrophages and activated ERK, JNK, and p38 contribute to the expression of inflammatory mediators [6]. Therefore, we also investigated the effect of flavonoids on LPS-induced phosphorylation of MAPKs in LPS-induced RAW 264.7 cells. The results showed that LPS activated MAPKs and that the levels of ERK1/2, JNK, and p38 phosphorylation decreased significantly following flavonoid treatment (Figures 4(a) to 4(a-2)). Interestingly, these results matched the reduced NF-*κ*B translocation and COX-2 and iNOS protein and mRNA expression results.

A RT-PCR analysis and ELISA were conducted using TNF-*α* and IL-6 primers and ELISA kit, respectively, to further investigate the effect of the flavonoids on LPS-induced production of proinflammatory cytokines. Proinflammatory cytokines are produced in macrophages activated by LPS and play a major role regulating inflammation and tumor progression [7]. Cytokines including TNF-*α* and IL-6 cause outbreaks of inflammatory disease associated with autoimmune disorders. These cytokines upregulate the production of acute phase reactants and the maintenance of proinflammatory cytokine activity gives rise to chronic inflammation [25]. Conditions requisite to increase NO production can result in the development of inflammatory diseases by TNF-*α*-stimulated IL-6 generation [6]. Accordingly, suppressing inflammatory cytokine production is a key mechanism to control inflammation. TNF-*α* and IL-6 expression was elevated following LPS-stimulation but their expression level decreased significantly in a dose-dependent manner following treatment with the flavonoids in mRNA and protein level (Figures 4(b) to 4(b-2)). Interestingly, this result matched with previous results including COX-2, iNOS mRNA, and protein expression levels and the NF-*κ*B and MAP kinase signal pathway result.

Furthermore, we conducted 2-DE and MALDI-TOF/MS analysis focusing on the protein expression pattern stimulated by LPS and flavonoid pretreatment of RAW 264.7 cells. Inflammatory response-related proteins such as the PRKA anchor protein and heat shock protein 70 kD were identified (Figure 5 and Table 1). Heat shock protein 70 kD is stimulated by cytokine, cellular stress, ultraviolet light, and constant heat exposure. Pretreatment with the flavonoids significantly decreased the level of heat shock protein 70 kD compared with that in the LPS treatment group, indicating that flavonoids prevented a cellular stress reaction.

## 5. Conclusion

We showed that flavonoids of *S. baicalensis *G. significantly curtailed production of inflammatory mediators (COX-2 and iNOS) at both the mRNA and protein levels and gene expression of proinflammatory cytokines (TNF-*α* and IL-6) in LPS-stimulated RAW 264.7 macrophage cells. In addition, the flavonoids markedly inhibited activation of NF-*κ*B and MAPK. These results support the anti-inflammatory effects of flavonoids in Korean *S. baicalensis *G. by suppressing NF-*κ*B and I*κ*B*α* phosphorylation and inhibiting NF-*κ*B translocation into the nucleus via downregulation of ERK1/2, JNK, and p38 signaling pathways. 

## Figures and Tables

**Table 1:** Identification of proteins regulated by flavonoids isolated from Korean *Scutellaria baicalensis  *Georgi.

Identified protein	Accession number	Score	Sequence coverage (%)/peptides matched	^ a^Theo. MW (Da)/pI	Mass values matched	Up or down
Protein disulfide-isomerase precursor	42415475	135	52	4.77	26	Down
Dynein heavy chain 10	254692843	52	4	5.53	20	Down
Heat shock 70 kD protein 5	148676670	81	29	5.03	13	Down
A kinase (PRKA) anchor protein	148682679	70	21	5.00	63	Down
Mitochondrial ATP synthase	89574015	68	24	4.90	9	Up
Pyruvate kinase isozymes	31981562	83	32	7.18	14	Down

^a^Theo. MW/pI, theoretical molecular weight and pI obtained from the NCBInr.
